# Protective Effect of CXCR4 Antagonist CX807 in a Rat Model of Hemorrhagic Stroke

**DOI:** 10.3390/ijms21197085

**Published:** 2020-09-25

**Authors:** Seong-Jin Yu, Kuo-Jen Wu, Yu-Syuan Wang, Jen-Shin Song, Chien-Huang Wu, Jiing-Jyh Jan, Eunkyung Bae, Hsi Chen, Kak-Shan Shia, Yun Wang

**Affiliations:** 1 Center for Neuropsychiatric Research, National Health Research Institutes, Zhunan 35053, Taiwan; b7508@nhri.edu.tw (S.-J.Y.); kjwu@nhri.edu.tw (K.-J.W.); yswang@nhri.edu.tw (Y.-S.W.); baee@nhri.edu.tw (E.B.); Lunachen@nhri.edu.tw (H.C.); 2 Institute of Biotechnology and Pharmaceutical Research, National Health Research Institutes, Zhunan 35053, Taiwan; jssong@nhri.edu.tw (J.-S.S.); markwu@nhri.edu.tw (C.-H.W.); jjjan@nhri.edu.tw (J.-J.J.); ksshia@nhri.edu.tw (K.-S.S.)

**Keywords:** hemorrhagic stroke, CXCR4, inflammation, protection

## Abstract

Intracerebral hemorrhage (ICH) is a major cause of stroke, with high mortality and morbidity. There is no effective pharmacological therapy for ICH. Previous studies have indicated that CXCR4 antagonists reduced microglia activation, attenuated infiltration of T cells, and improved functional recovery in ischemic stroke animals. The interaction of CXCR4 antagonists and ICH has not been characterized. The purpose of this study is to examine the neuroprotective action of a novel CXCR4 antagonist CX807 against ICH. In primary cortical neuronal and BV2 microglia co-culture, CX807 reduced glutamate-mediated neuronal loss and microglia activation. Adult rats were locally administered with collagenase VII to induce ICH. CX807 was given systemically after the ICH. Early post-treatment with CX807 improved locomotor activity in ICH rats. Brain tissues were collected for qRTPCR and histological staining. ICH upregulated the expression of CXCR4, CD8, TNFα, IL6, and TLR4. The immunoreactivity of IBA1 and CD8, as well as TUNEL labeling, were enhanced in the perilesioned area. CX807 significantly mitigated these responses. In conclusion, our data suggest that CX807 is neuroprotective and anti-inflammatory against ICH. CX807 may have clinical implications for the treatment of hemorrhagic stroke.

## 1. Introduction

Intracerebral hemorrhage (ICH) is a major cause of stroke, with high mortality and morbidity. Current treatment focuses on controlling the bleeding, removing the blood clot, and relieving intracranial pressure caused by bleeding in the brain. There is no effective therapy for ICH. After the onset of ICH, a series of neurodegenerative and inflammatory cascade reactions are turned on. These include disruption of the blood–brain barrier, upregulation of chemokines, activation of microglia, mobilization of peripheral lymphocytes to the lesioned brain, apoptosis, and cell death [1]. As inflammation is closely associated with degeneration in the brain, anti-inflammatory agents may reduce brain damage and improve outcomes in ICH patients.

Chemokines are critical mediators for neuroinflammation. These ligands regulate microglia activation and leukocyte migration to the injured brain. C-X-C motif ligand 12 (CXCL12 or SDF-1α) is a pleiotropic chemokine which constitutively expresses in the central nervous system and periphery. CXCL12 interacts with its receptor CXCR4 (C-X-C chemokine receptor type 4). CXCR4 is expressed in immune and hemopoietic cells. It is also present in neurons, astrocytes, microglia [2], bone marrow-derived cells, and neural progenitor cells [3,4,5]. CXCL12 triggers the migration of CXCR4 (+) inflammatory cells to the lesioned site after brain injury. Enhanced CXCR4 and microglia immunoreactivities were found in the penumbra of the ischemic brain [2]. Similarly, the expression of CXCR4 mRNA was upregulated at 2–4 days after middle cerebral artery occlusion [4,6]. CXCR4 antagonists CX549 or AMD3100 reduced microglia activation [7,8] and T cell infiltration in the ischemic hemisphere and facilitated functional recovery in stroke animals [8,9,10]. These data suggest that ischemic brain injury enhances inflammation through CXCR4. The physiological role of CXCL12/CXCR4 in the ICH brain, however, is still not clear. One study indicated that ICH patients had higher serum CXCL12 levels than healthy controls. The neurological score and hematoma volume were positively correlated with CXCL12 levels in ICH patients [11]. These findings support the notion that ICH activates CXCL12/CXCR4 cascade. The use of selective CXCR4 antagonists may thus attenuate ICH-mediated CXCL12 chemotactic action and brain damage.

**Figure 1:**
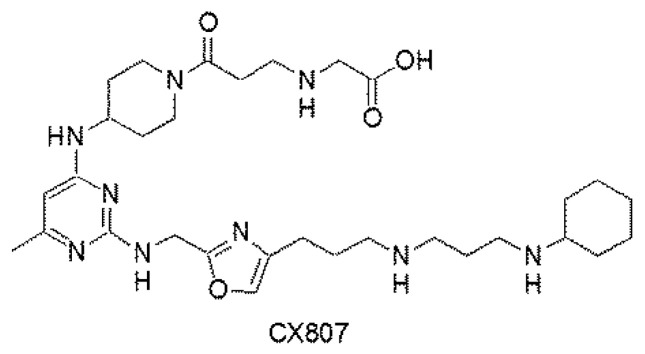
Chemical structure of CX807.

The purpose of this study was to characterize the neuroprotective and anti-inflammatory effects of a novel CXCR4 antagonist CX807 (Figure 1) in cell culture and a rodent model of ICH. Our data support the notion that CX807 is a potent anti-inflammatory agent and early post-treatment with CX807 protects against ICH -mediated neurodegeneration.

## 2. Results

### 2.1. In Vitro and In Vivo Characterization of CX807 and AMD3100

We first compared the binding affinity of CX807 and a commonly used CXCR4 antagonist AMD3100 (or Plerixafor) in the purified membrane fractions of HEK293 cells overexpressing CXCR4 [8]. CX807 and AMD3100 displaced [^125^I]CXCL12 binding to CXCR4 at the IC_50_ of 65.4 ± 11.8 nM and 146.7 ± 30.7 nM, respectively. The chemotactic action of CXCL12 was examined in cultured CCRF-CEM cells, a human cell line with high CXCR4 expression [8]. Similar to the binding assay, CX807 was more potent than AMD3100 at inhibiting CXCL12-mediated chemotaxis (EC_50_: 43.9 ± 11.3 nM for CX807 vs. 76.9 ± 11.6 nM for AMD3100). The specificity of CX807 was analyzed by a β-arrestin assay [8]. CX807, at a 10 μM dose, selectively antagonized binding to CXCR4, but not to the other 16 chemokine receptors. These data support the notion that CX807 has a high affinity and selectivity to the CXCR4 and is efficient in suppressing CXCL12-mediated chemotaxis in vitro.

The pharmacokinetic profile of CX807 was examined in C57BL/6 mice (*n* = 3). Blood samples were collected at 0.03, 0.08, 0.25, 0.5 1, 2, and 4 h after systemic administration of CX807 (6 mg/kg, i.p.). Plasma CX807 level peaked (Cmax = 11,953 ng/mL) at 0.25 h. The estimated t1/2 was 0.63 h, and AUC was 10,953 ng/mL × h. 

### 2.2. CX807-Mediated Neuroprotection in Primary Cortical Cells and Microglia Co-Cultures

**Figure 2:**
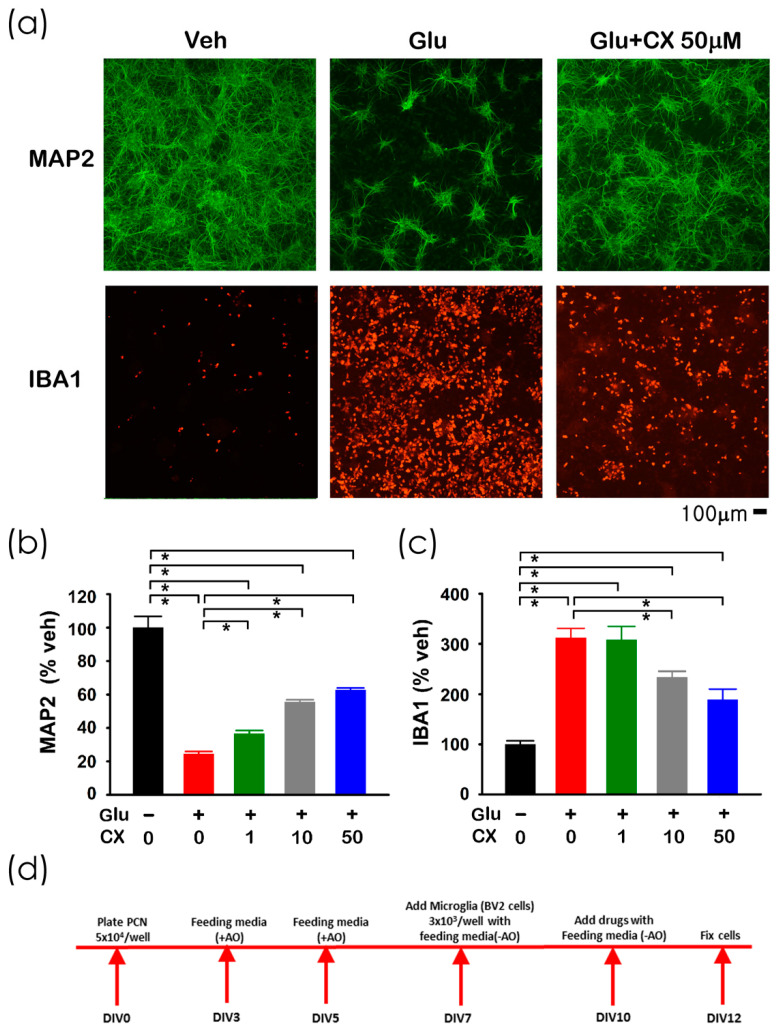
CX807 antagonized Glu-mediated neuronal loss and microglia activation in primary cortical neuronal and BV2 microglial co-culture. (**a**) Treatment with glutamate (Glu) reduced MAP2 (in green color) while increasing IBA1 (red) immunoreactivity in microglia and neurons co-culture. CX807 antagonized both responses. Calibration = 100 µm. Immunoreactivity of MAP2 (**b**) and IBA1 (**c**) were normalized to the mean of the control samples. CX807 (1–50 µM) significantly antagonized glutamate (100 µM)-mediated changes in MAP2 and IBA1 immunoreactivity. (**d**) Timeline of the experiment. Data are represented as mean +/− SEM. * *p* < 0.05, 1-Way ANOVA on rank + post-hoc NK test.

Glutamate (Glu), CX807, or vehicle were added to the primary cortical neuronal and BV2 microglial co-culture [12] on DIV10. Cells were fixed for immunocytochemistry on DIV12 (see timeline, Figure 2d). A high dose (100 µM) of Glu was used to generate neurodegeneration and inflammation [12] and to simulate overflow of elevated glutamate in stroke [13,14]. Glutamate significantly reduced the neuronal marker microtubule-associated protein 2 (MAP2; Figure 2a,b, *p* < 0.001) while increased microglia marker ionized calcium-binding adapter molecule 1 (IBA1) immunoreactivity (Figure 2a,c, *p* < 0.001). Both responses were dose-dependently mitigated by CX807 (Figure 2b, MAP2, *p* < 0.001; Figure 2c, IBA1: *p* = 0.005).

### 2.3. CX807 Improved Locomotor Behavior in ICH Rats

**Figure 3:**
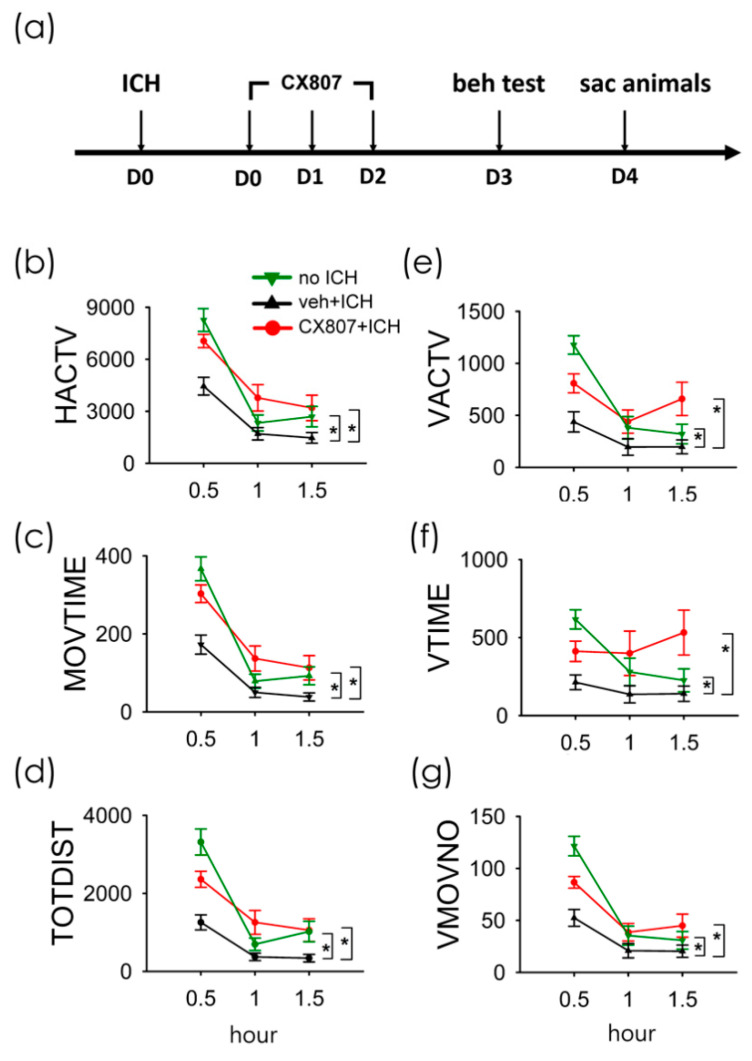
Treatment with CX807 normalized locomotor behaviors in ICH rats. (**a**) Timeline of in vivo experiment. Adult rats received intrastriatal administration of collagenase type VII to induce ICH on D0. CX807 or vehicle was given after ICH from D0 to D2. The locomotor behavior was recorded for 90 min on D3. ICH significantly reduced horizontal movement (veh + ICH vs. no ICH, (**b**): HACTV, (**c**): MOVTIME; (**d**): TOTDIST) and vertical movement ((**e**): VACTV, (**f**): VTIME, (**g**): VMOVNO). The reduction of locomotor activity after ICH injury was significantly antagonized by post-treatment with CX807. HACTV = horizontal activity; MOVTIME = horizontal movements time in second; TOTDIST = total distance traveled in centimeters; VACTV = vertical activity; VTIME = vertical movements time in second; VMOVNO = number of vertical movements. * *p* < 0.001, two-way ANOVA + post-hoc NK test. Data are represented as mean +/− SEM.

A total of 38 rats were used for the behavioral assay. Animals were separated into three groups (no ICH, *n* = 11; ICH+veh, *n* = 15; ICH + CX807, *n* = 12). The ICH rats received intra-striatal administration of collagenase type VII (0.5 unit/µL × 2µL). CX807 (3 mg/kg/d × 3 days ICH + CX807) or vehicle (ICH + veh) was administered i.p. from day 0 to day 2 (see timeline, Figure 3a). Animals were placed in infra-red behavioral chambers for 90 min for locomotor behavioral analysis on D3 (Figure 3a). As seen in Figure 3 and Table 1, ICH significantly reduced horizontal and vertical movement (*p* < 0.001, veh + stroke vs. no stroke, two-way ANOVA). CX807 significantly improved locomotor function (Table 1 and Figure 3, *p* < 0.001, CX807 + stroke vs. veh + stroke). No significant difference was found between the ICH + CX807 and no ICH groups over the entire 90 min period (Table 1). Post-hoc NK analysis indicated that CX807 partially improved locomotor activity in the first 30 min in ICH rats (CX807 + ICH vs. no ICH: MOVTIME, *p* < 0.001; TOTDIST, *p* = 0.004; VACTV, *p* = 0.015; VMOVNO, *p* = 0.005, Figure 3).

### 2.4. CX807 Reduced ICH-Mediated Expression of CXCR4, TLR4, IL6, CD8, and TNFα

**Figure 4:**
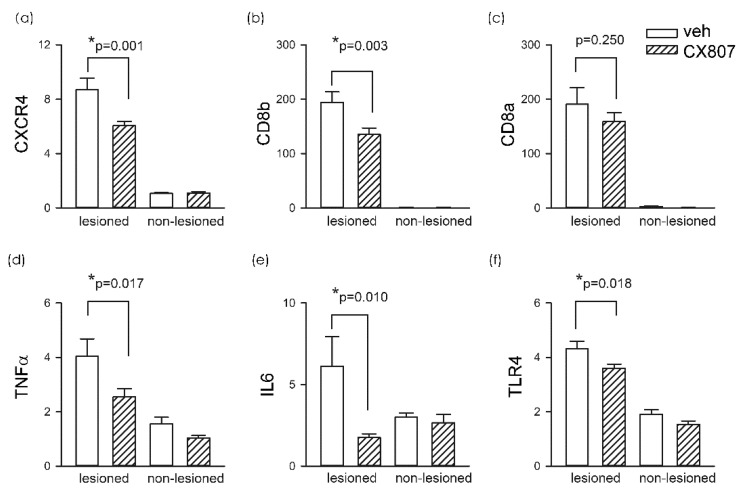
CX807 reduced the expression of inflammatory markers. The expression of the target genes was examined by qRTPCR and normalized relative to the endogenous reference gene (beta-actin and GAPDH averages) with a modified delta-delta-Ct algorithm. ICH significantly increased the expression of (**a**) CXCR4, (**b**) CD8a, (**c**) CD8b (**d**) TNFα, (**e**) IL6, and (**f**) TLR4 in the lesioned side striatum, as compared to the non-lesioned side. Treatment with CX807 reduced CXCR4, TLR4, CD8b. TNFα and IL6 expression. *p values were calculated using a 2-way ANOVA + post-hoc NK test.

A total of 19 rats received intrastriatal administration of collagenase type VII followed by daily treatment with vehicle (*n* = 11) or CX807 (*n* = 8). Striatal tissues were collected on day 4 for qRTPCR analysis. In animals receiving vehicle, ICH significantly increased CXCR4 mRNA in the lesioned side striatum, as compared to the non-lesioned side (*p* < 0.001, 2-way ANOVA + NK test). ICH also increased the expression of toll-like receptor 4 (TLR4, *p* < 0.001), CD8a (*p* < 0.001), and CD8b (*p* < 0.001). The changes in microglia and T cell markers were associated with significant upregulation of proinflammatory cytokines TNFα (*p* < 0.001) and IL6 (*p* = 0.046) (Figure 4). These inflammatory markers in the lesioned striatum were mitigated after CX807 treatment. CX807 significantly reduced CXCR4 (Figure 4, *p* = 0.001, CX807 + ICH vs. veh + ICH, 2-way ANOVA + NK test), TLR4 (*p* = 0.018, CX807 + ICH vs. veh + ICH), and CD8b (*p* = 0.003, CX807 + ICH vs. veh + ICH). There is a non-significant trend that CX807 downregulated CD8a in the ICH brain. CX807 also significantly attenuated TNFα (*p* = 0.017, CX807 + ICH vs. veh + ICH) and IL6 (*p* = 0.010, CX807 + ICH vs. veh + ICH) expression in the lesioned striatum (Figure 4).

### 2.5. CX807 Mitigated IBA1, CD8 Immunoreactivity, and TUNEL in ICH Brains

**Figure 5:**
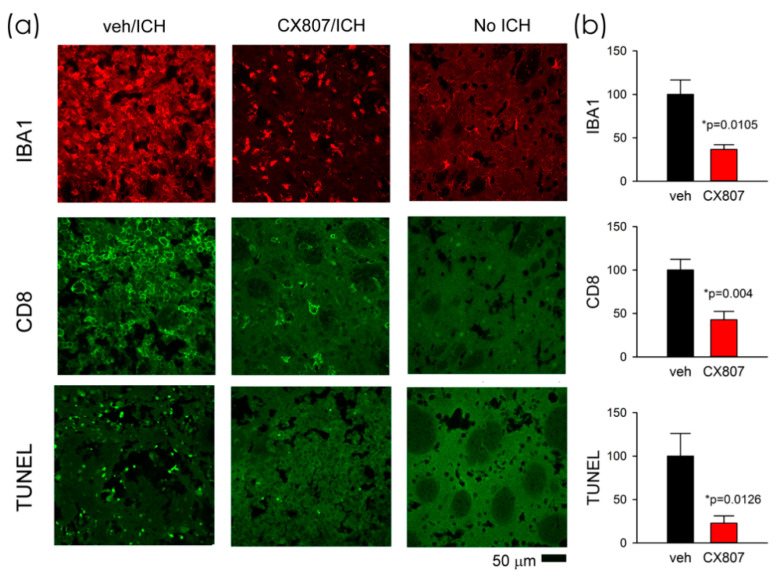
CX807 mitigated IBA1 and CD8 immunoreactivity as well as TUNEL in the ICH brain. (**a**) Representing photomicrographs were taken from the perilesioned area of ICH animals receiving veh (veh/ICH) or CX807 (CX807/ICH). ICH increased TUNEL, IBA1, and CD8 fluorescence, which were mitigated by CX807. No TUNEL was found in the contralateral side striatum (No ICH). (**b**) TUNEL, IBA1, and CD8 activities were averaged from 6 regions (1267 × 1267 µm) of two adjacent brain slices at the level of anterior commissure from each brain. CX807 significantly reduced TUNEL, IBA1, and CD8 optical density. Data are represented as mean +/− SEM. *p values were calculated using a *t*-test.

Animals were sacrificed on day 4 for histological analysis. TUNEL staining was conducted in eight ICH rats. Collagenase injection resulted in hematoma, necrosis, or tissue loss near the core of the injection. The injured area was surrounded by TUNEL (+) cells. As seen in the representing photomicrographs, ICH increased TUNEL labeling in the perilesioned area (Figure 5a, veh/ICH vs. No ICH). CX807 mitigated TUNEL in the lesioned brain (Figure 5, CX807/ICH vs. veh/ICH). Enhanced IBA1 and CD8 immunoreactivities were also found in the lesioned striatum; CX807 antagonized these responses (Figure 5). TUNEL, IBA1, and CD8 activities in the perilesioned area were quantified. The optical density was averaged from six images (each 1267 × 1267 µm^2^) in brain slices with visible anterior commissure for each animal. CX807 significantly reduced TUNEL (*p* = 0.0126, *t*-test), IBA1 (*p* = 0.0105), and CD8 (*p* = 0.004) in the perilesioned area (Figure 5b). 

## 3. Discussion

**Figure A1:**
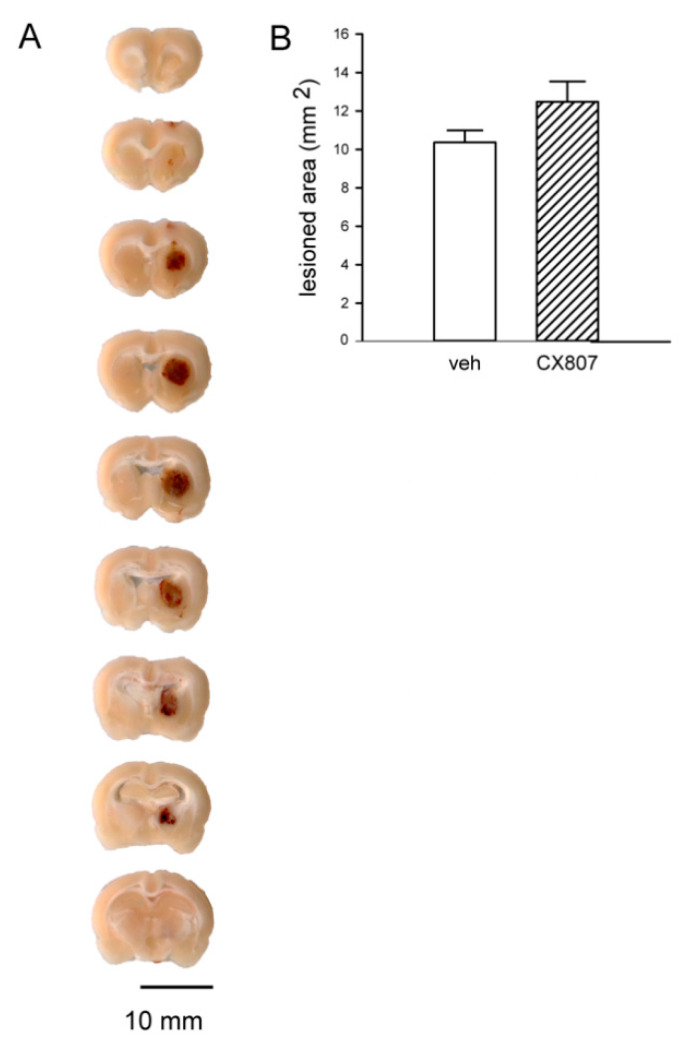
Intracerebral administration of collagenase induced ICH in striatum. (**A**) Brain tissue was collected, sectioned (at 1-mm thickness), and PFA -fixed at 4 days after administration of collagenase (0.5 unit/μL × 2 μL). Cerebral hemorrhage (brown color) was found in the ipsilateral striatum. (**B**) Brain samples were also cut at a 30-μm thickness on a cryostat. Slices were rinsed in PB, mounted on slides, and cover-slipped. The area of lesion (tissue loss after rinsing with PB) was analyzed in brain sections with visible anterior commissure. CX807 did not significantly alter the lesioned area (*p* = 0.204, *t* test).

We demonstrated that local collagenase injection resulted in intracerebral hemorrhage (Figure A1 in Appendix A) and bradykinesia in rats. Early post-treatment with a novel CXCR4 antagonist CX807 improved locomotor activity. ICH upregulated the expression of inflammatory and apoptotic markers, including TLR4, TNFα, IL6, CD8, and TUNEL, in the lesioned brain. CX807 significantly antagonized these responses. The main finding of this study is that CX807 reduced neuroinflammation and neurodegeneration in ICH rats.

We characterized the binding and function of CX807 in vitro. Compared to a commonly used CXCR4 antagonist, AMD3100, CX807 displaced CXCL12 binding at lower concentrations and was more efficient at inhibiting CXCL12-mediated chemotaxis. In the β-arrestin assay, we demonstrated that CX807 selectively antagonized binding to CXCR4. These data suggest that CX807 is a CXCR4 antagonist with high affinity and specificity. 

The interaction of CXCL12/CXCR4 and neuroinflammation has been studied in cell culture. Lipopolysaccharide increased CXCL12 expression in primary cultured microglia [15]. CXCL12 induced the proliferation of microglia through the activation of Erk1/2 and Akt signaling [2,16]. In hippocampal slice and astrocytic culture, CXCL12 stimulated glutamate release, which was further amplified by reactive microglia and reduced by AMD3100 [17]. These data suggest that CXCL12/CXCR4 reciprocally activates the inflammatory response. In this study, CX807 dose-dependently attenuated glutamate-mediated inflammation and neurotoxicity in neuron/microglia co-culture. CX807 attenuated IBA1 immunoreactivity and restored neuronal marker MAP2 after injury. Our data support the notion that CXCR4 antagonist CX807 is anti-inflammatory and neuroprotective in vitro. 

**Figure A2:**
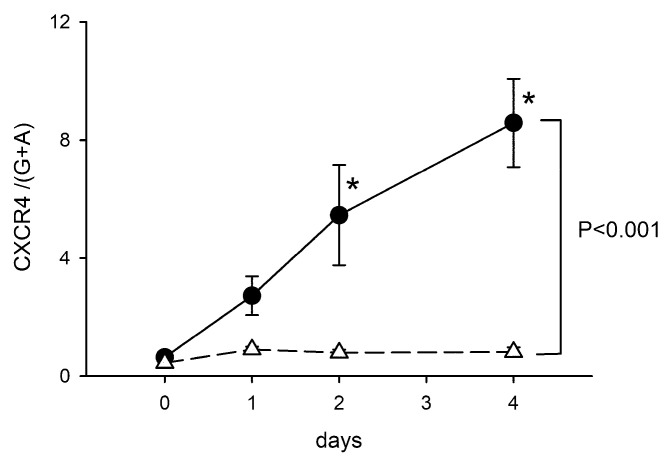
Time-dependent upregulation of CXCR4 after intrastriatal administration of collagenase. The expression of CXCR4 mRNA in the lesioned striatum was significantly increased from D1 to D4. Data for D0 were taken from non-ICH control animals. The expression of CXCR4 was normalized relative to the endogenous reference genes (Beta-actin and GAPDH averages) with a modified delta-delta-Ct algorithm. * *p* < 0.05, F_3,25_ = 5.990, vs. control, Two-way ANOVA + posthoc NK test.

ICH was introduced, in the current study, by intracerebral administration of collagenase [18]. After the injury, ICH induced time-dependent expression of chemokine receptor CXCR4, which increased from D1 to D4 (Figure A2 in Appendix A). Based on the expression profile of CXCR4, CX807 was applied early after the injury from D0 and D2. The behavior was examined on D3, and brain tissues were collected on D4 (Figure 3a). We demonstrated that CX807 significantly improved locomotor movements in ICH rats. A similar behavioral response was found in animals receiving AMD3100 (data not shown). Both AMD3100 and CX807 normalized horizontal and vertical locomotor function in ICH rats. CX807 was more potent than AMD3100 at improving vertical movement time. 

In a pilot study to examine the optimal dose for lesioning, collagenase (0.5 unit/μL × 1 μL or 2 μL) was injected into the striatum of adult rats. Animals receiving high-dose collagenase (i.e., 0.5 unit/μL × 2 μL) produced consistent behavioral and biochemical changes. This dose was later used to examine the protective action of CX807 in a controlled and randomized study. The data from the pilot (0.5 unit/μL × 2 μL collagenase) study were included for statistics, as it was seen that more animals were used for ICH + veh than ICH + CX807 in behavior and qRTPCR analysis.

Increasing evidence has supported the notion that the release of pro-inflammatory molecules from immune cells contributes to neurodegeneration. We demonstrated that ICH upregulated proinflammatory IL6 and TNFα as well as TUNEL. These responses were significantly attenuated by CX807, suggesting that CX807 suppressed ICH -mediated inflammation and cell death in the lesioned brain. 

Peripheral immune response contributes to neuroinflammation in stroke brain. For example, splenectomy or systemic administration of minocycline reduced infarction and microglia activation in the ischemic brain [19,20]. Similar findings were reported in the current study. ICH significantly increased the expression of cytotoxic T cell marker CD8, suggesting the presence of peripheral immune cells in the lesioned brain. As CXCL12 modulated the migration of T cells, B cells, and monocytes across the blood–brain barrier [21], CXCR4 antagonist CX807 was used to attenuate peripheral inflammation. Post-treatment with CX807 significantly reduced ICH-mediated CD8 expression. CXCL12 also upregulated innate inflammation through CXCR4 dependent pathway in microglia [22]. Activation of TLR4 after injury leads to inflammatory cytokine production and initiates the innate immune response [23]. TL4R and microglia marker IBA1 were upregulated after hypoxic or ischemic brain injuries [23,24]. In this study, ICH increased TLR4 and IBA1 expression, which was significantly antagonized by CX807. Taken together, our data support the notion that CX807 suppresses T-cell migration from the periphery and innate microglia activation in the ICH brain. 

In a preliminary radioligand binding assay, we found that CX807, at a high dose, partially inhibited bradykinin B1 receptor (B1R) binding. It has been shown that B1R antagonist mitigated hemorrhage, improved neurobehavioral deficits, and preserved blood–brain barrier integrity after reperfusion [25]. CX807 may reduce ICH damage partially through the B1R, which warrants further investigation.

In conclusion, our data support the notion that early post-treatment with CXCR4 antagonist CX807 reduces ICH-mediated inflammation and apoptosis. Previous studies have indicated that CXCR4 antagonists reduced brain degeneration and improved behavioral recovery in the animals with ischemic stroke [7,8,9,26]. A common mechanism involving CXCR4 may contribute to neurodegeneration in hemorrhage and ischemic stroke. CXCR4 antagonists may be useful in treating both types of stroke in patients.

## 4. Materials and Methods 

### 4.1. Animals and Material 

Adult male Sprague–Dawley rats (ICH study) and C57BL/6 mice (for pharmacokinetic study) were used for this study. The use of animals was approved by the Animal Care and Use Committee of the National Health Research Institute, Taiwan (105080-A, 2016-05; 108146-A, 2020-1). All animal experiments complied with the Animal Research: Reporting of In Vivo Experiments (ARRIVE) guidelines and were carried out in accordance with the National Institutes of Health guide for the care and use of laboratory animals (NIH Publications No. 8023, revised 1978). CX807 was supplied by Dr. K.-S. Shia at the National Health Research Institutes, Taiwan.

### 4.2. CXCR4-Binding Assay

Aliquots of 2.5 μg of purified HEK293T cell membrane fractions with CXCR4 were incubated with 0.16 nM [^125^I]CXCL12 (PerkinElmer, Waltham, MA, USA) and the indicated concentrations of BPRCX807 in incubation buffer (50 mM HEPES-NaOH, pH 7.4, 100 mM NaCl, 5 mM MgCl_2_, 1 mM CaCl_2_, 0.5% BSA). Nonspecific binding was defined in the presence of 50 μM AMD3100 (plerixafor). The reaction mixtures were incubated for 1.5 h at 30 °C and then transferred to a 96-well GF/B filter plate (Merck Millipore, Billerica, MA, USA). The reaction mixtures were terminated by manifold filtration and washed with ice-cold wash buffer (50 mM HEPES-NaOH, pH 7.4, 100 mM NaCl) four times. The radioactivity bound to the filter was measured with a TopCount instrument (PerkinElmer, Waltham, MA, USA). The IC_50_ values were determined as the concentrations of the compounds required to inhibit 50% of the specific binding of [^125^I]CXCL12 and were calculated by nonlinear regression (GraphPad software, San Diego, CA, USA).

### 4.3. CXCL12-Induced Chemotaxis Assay

CCRF-CEM (T-cell acute lymphoblastic leukemia) cells were suspended in RPMI 1640 containing 10% FBS and then preincubated with compounds for 10 min at 37 °C. The assay was performed in Millicell Hanging Cell Culture Inserts (pore size 5 μm; 24-well plate; Millipore, Bedford, MA, USA). CX807 containing 10 nM recombinant CXCL12 (PeproTech, Rocky Hill, NJ, USA) was plated in the lower chambers of inserts, and cells with compounds were plated in the upper chambers of inserts at a density of 2.5 × 10^5^ cells/well. After 2.5 h incubation at 37 °C, cells in both chambers of inserts were incubated for an additional 1.5 h with PrestoBlue cell viability reagent, according to the manufacturer’s instructions, from Invitrogen (Waltham, MA, USA).

### 4.4. β-Arrestin Functional Assay

β-Arrestin functional assay [27] was conducted by the Eurofins DiscoverX (Fremont, CA, USA). The PathHunter^®^ β-Arrestin cell lines expressing chemokine receptors were used. The functional reporter β-galactosidase (β-Gal) was split into two complementary fragments: a small peptide (ProLink™, PK) and a larger protein (Enzyme Acceptor, EA). PK was tagged with G-protein coupled receptor (PK/GPCR), while EA was tagged with β-Arrestin (β-Arrestin/EA). Activation of receptors following ligand binding recruited β-Arrestin/EA to the PK/GPCR, resulting in PK and EA complementation, and restored β-Gal activity. The enzymatic activity of β-Gal was measured using chemiluminescent PathHunter^®^ Detection Reagents. For antagonist determination, cells were pre-treated with 10 μM CX807 followed by chemokine challenge. 

### 4.5. Pharmacokinetic Analysis and Toxicology Study

Male C57BL/6 mice, each weighing 23.4–25.4 g, were quarantined for 1 week before drug treatment. CX807, dissolved in 100% saline, was given to mice (i.p.). Animals were anesthetized by isoflurane. Blood samples were collected via cardiac puncture at defined time points 0, 0.03, 0.08, 0.25, 0.5, 1, 2, and 4 h after dosing and then stored at −80°C. The volume of the dosing solution given was 100 μL for each mouse. The plasma samples were analyzed by liquid chromatography–tandem mass spectrometry (LC-MS/MS), and the data were calculated by a standard noncompartmental method using the Kinetica software program (InnaPhase, Philadephia, PA, USA). 

### 4.6. Primary Rat Cortical Neuron (PCN) and Microglia Co-Culture 

Primary cortical neuron (PCN) cultures (around 65% neurons + 35% glia) were prepared from embryonic (E14–15) cortex tissues obtained from fetuses of timed pregnant Sprague–Dawley rats. After removing the blood vessels and meninges, pooled cortices were trypsinized (0.05%; Invitrogen, Carlsbad, CA, USA) for 20 min at room temperature. After rinsing off trypsin with pre-warmed Dulbecco’s modified Eagle’s medium (Invitrogen, Carlsbad, CA, USA), cells were dissociated by trituration, counted, and plated into 96-well (5.0 × 10^4^/well) cell culture plates pre-coated with poly -D-Lysine (Sigma-Aldrich, St. Louis, MO, USA). The culture plating medium consisted of neurobasal medium supplemented with 2% heat-inactivated FBS, 0.5 mmol/L L-glutamine, 0.025 mM L-glutamate, and 2% B27 (Invitrogen, Carlsbad, CA, USA). Cultures were maintained at 37 °C in a humidified atmosphere of 5% CO_2_ and 95% air. The cultures were fed by exchanging 50% of media with feed media (neurobasal medium) with 0.5 mM L-glutamine, and 2% B27 with antioxidant supplement on days in vitro (DIVs) 3 and 5. 

BV2 microglia were cultured separately, detached by 0.05% trypsin-ethylene diamine tetraacetic acid (EDTA, Invitrogen), and centrifuged at 100 g for 5 min. BV2 cells were resuspended in the feeding media containing B27 supplement without antioxidants (-AO, from Invitrogen, Carlsbad, CA, USA). The density of surviving cells was counted using a trypan blue assay; cells were plated on the PCN plated wells at a concentration of 3.0 × 10^3^/well on DIV 7, as we previously described [12]. The co-cultures were fed with –AO media on DIVs 7 and 10. On DIV 10, cultures were treated glutamate CX807 or vehicle. At 48 h after drug treatment, cells were fixed with 4% paraformaldehyde (PFA, Sigma-Aldrich, St. Louis, MO, USA) for 1 h at room temperature.

### 4.7. Immunocytochemistry

After removing 4% PFA solution, cells were washed with PBS. Fixed cells were treated with a blocking solution (5% BSA and 0.1% Triton X-100 in PBS) for 1 h. The cells were incubated for 1 day at 4 °C with a mouse monoclonal antibody against MAP2 (1:500; Millipore, Billerica, MA, USA) and rabbit polyclonal antibody against IBA1 (1:500; Wako, Richmond, VA, USA) and then rinsed three times in PBS. The bound primary antibody was visualized using AlexaFluor 488 goat anti-mouse or AlexFluoro 568 goat anti-rabbit secondary antibody (Invitrogen, Carlsbad, CA, USA). Images were acquired using a camera DS-Qi2 (Nikon, Tokyo, Japan) attached to a NIKON ECLIPSE Ti2 (Nikon, Tokyo, Japan) inverted microscope by blinded observers. Data were analyzed using NIS Elements AR 5.11 Software (Nikon).

### 4.8. Collagenase-Induced Intracerebral Hemorrhage 

Rats were housed in a 12-h dark (7 pm to 7 am) and 12-h light (7 am to 7 pm) cycle. Animals were anesthetized and placed in a stereotaxic frame. Type VII collagenase (0.5 U/μL × 2.0 μL, C-0773, Sigma Aldrich, St. Louis, MO, USA) was stereotactically injected into the right striatum (coordinates: 0.0 mm rostral and 3.0 mm lateral to bregma, 5.5 mm below the skull) at 0.4 μL/min over 5 min on day 0. CX807 (3 mg/kg/d × 3 days) or vehicle was administered i.p. from day 0 to day 2. Animals were sacrificed on day 4 for histological and PCR analysis.

### 4.9. Locomotor Behavioral Measurement 

Locomotion was measured on day 3 using an infrared activity monitor (Accuscan, Columbus, OH). Rats were individually placed in a 3D infrared behavior chamber (42 × 42 × 21 cm) for 90 min. Six variables were measured: (i) horizontal activity (HACTV, the total number of beam interruptions that occurred in the horizontal sensors), (ii) horizontal movements time (MOVTIME), and (iii) total distance traveled (TOTDIST, the distance, in centimeters, traveled by the animals), (iv) vertical activity (VACTV, the total number of beam interruptions that occurred in the vertical sensors), (v) vertical movements time (VTIME), and (vi) number of vertical movements (VMOVNO).

### 4.10. Immunohistochemistry 

Animals were anesthetized and perfused transcardially with saline followed by 4% PFA in phosphate buffer (PB; 0.1 mol/L; pH 7.2); they were post-fixed for 18–20 h and then transferred to 20% sucrose in 0.1 M PB for at least 16 h. Serial sections of brains were cut at a 30-μm thickness on a cryostat (model: CM 3050 S; Leica, Heidelberg, Germany). Brain sections were rinsed in PB and were blocked with 4% bovine serum albumin (Sigma-Aldrich) with 0.3% Triton X-100 (Sigma-Aldrich) in 0.1 mM PB. Brain slices were then incubated with primary antibodies against CD8 (monoclonal 1:100, Abcam, Cambridge, UK) or IBA1 (monoclonal 1:100, Chemicon, Billerica, MA, USA) at 4 °C overnight. Sections were rinsed in 0.1 mM PB and incubated in Alexa Fluor 488 secondary antibody solution (1:500; Molecular Probes, Eugene, OR, USA). Control sections were incubated without the primary antibody. Brain sections were mounted on slides and coverslipped. Confocal analysis was performed using a Nikon D-ECLIPSE 80i microscope (Nikon Instruments, Inc., Tokyo, Japan) and EZ-C1 3.90 software (Nikon, Tokyo, Japan). The optical density of IBA1 or CD8 immunoreactivity was quantified in two consecutive brain sections with a visualized anterior commissure in each animal. Two photomicrographs were taken along the perilesioned region per brain slice; IBA1 or CD8 optical density was analyzed by NIS Elements AR 3.2 Software (Nikon) and was averaged in each brain for statistical analysis. All immunohistochemical measurements were performed by blinded observers.

### 4.11. TUNEL Labeling

Apoptotic cell death was detected by terminal deoxynucleotidyl transferase (TdT), which catalyzes the polymerization of labeled nucleotides to free 3′-OH DNA ends in a template-independent manner (TUNEL reaction), according to the manufacturer’s protocol (in situ cell death detection kit, Cat. No. 11684795910, Roche). Brain sections were attached to a glass slide and were air-dried at room temperature for 20 min. Sections were then washed with 0.1M PB for 30 min and incubated in permeabilization solution for 2 min on ice. Then, 50 µL of TUNEL reaction solution was added to the samples, and incubated samples were placed in a humidified atmosphere for 60 min at 37 °C in the dark. Sections were rinsed in 0.1 M PB and were coverslipped. For negative control, sections were incubated with the same volume of label solution instead of the TUNEL reaction solution. Confocal analysis was performed using a Nikon D-ECLIPSE 80i microscope (Nikon Instruments, Inc., Tokyo, Japan) and EZ-C1 3.90 software. The optical density of TUNEL positive cells was quantified in three consecutive brain sections with a visualized anterior commissure in each animal. Six photomicrographs were taken along the perilesioned region per brain slice; TUNEL positive optical density was analyzed by NIS Elements AR 3.2 Software (Nikon) and was averaged in each brain for statistical analysis.

### 4.12. Quantitative Reverse Transcription PCR (qRTPCR)

Striatal tissues from the lesioned and non-lesioned side hemispheres were collected. Total RNAs were isolated using an RNeasy Mini Kit (Qiagen, #74106, Germantown, MD, USA), and cDNAs were synthesized from 1 µg total RNA by use of RevertAid H Minus First Strand cDNA Synthesis Kit (Thermo Scientific, #K1631, Waltham, MA, USA). cDNA levels for CXCR4, CD8b, CD8a, TNFα, IL-6, TLR4, actin, and GAPDH were determined by). cDNA levels for CXCR4, CD8b, CD8a, TNFα, IL-6, TLR4, actin, and GAPDH were determined by specific universal probe library primer-probe sets or gene-specific primers (Table 2). Samples were mixed with TaqMan Fast Advanced Master Mix (Life Technologies, #4444557, Carlsbad, CA, USA) or SYBR (Luminaris Color HiGreen Low ROX qPCR Master Mix; ThermoScientific, Waltham, MA, USA). Quantitative real-time PCR (qRT-PCR) was carried out using the QuantStudio™ 3 Real-Time PCR System (ThermoScientific, Waltham, MA, USA). The expression of the target genes was normalized relative to the endogenous reference gene (beta-actin and GAPDH averages) with a modified delta-delta-Ct algorithm. All experiments were carried out in duplicate.

### 4.13. Statistics 

Data are presented as mean ± s.e.m. Unpaired *t*-test or 1 or 2-way ANOVA were used for statistical comparisons, with a significance level of *p* < 0.05. In the event of multiple comparisons, a post-hoc Newman–Keuls test (NK test) was performed.

## 5. Patents

CX807 has been filed for the USA patent application by Shia et al.

## Figures and Tables

**Table 1:** Significant differences in locomotor behaviors amongst all groups.

	*p*-Value			*F* Value
	veh + ICH vs.	CX807 + ICH vs.		*F*_2,105_ =
	no ICH	veh + ICH	no ICH	
HACTV	<0.001	<0.001	0.592	15.569
MOVTIME	<0.001	<0.001	0.803	18.172
TOTDIST	<0.001	<0.001	0.524	20.988
VACTV	<0.001	<0.001	0.916	13.322
VTIME	0.003	<0.001	0.311	9.928
VMOVNO	<0.001	<0.001	0.401	13.499

*p* and *F* values were calculated using a 2-way ANOVA and post-hoc NK test.

**Table 2:** Oligonucleotide primers used for quantitative RT-PCR.

Gene	SYBR Green		TagMan
	Forward	Reverse	
CXCR4	ATCATCTCCAAGCTGTCACACTCC	GTGATGGAGATCCACTTGTGCAC	
CD8a	ACACTTCGCAAGGATGCTCT	GTTGCTGGTGATTGAGCAGA	
CD8b	GTCTTTGGGACAGGGACAAA	GAGGGATACCAGCAGAACCA	
TNF-α	CCACACCGTCAGCCGATT	TCCTTAGGGCAAGGGCTCTT	
IL-6	GACCAAGACCATCCAACT	TAGGTTTGCCGAGTAGAC	
TLR4	AGCTTTGGTCAGTTGGCTCT	CAGGATGACACCATTGAAGC	
β-Actin			Rn00667869_m1
GAPDH			Rn01775763_g1

## Abbreviations

β-Gal	β-galactosidase
CXCL12	C-X-C motif ligand 12
DIVs	days in vitro
EDTA	ethylene diamine tetraacetic acid
HACTV	horizontal activity
IBA1	ionized calcium-binding adapter molecule 1
ICH	intracerebral hemorrhage
MAP2	microtubule-associated protein 2
MOVTIME	horizontal movements
NK test	Newman–Keuls test
PCN	primary cortical neuron
TLR4	toll-like receptor 4
TOTDIST	total distance traveled
VACTV	vertical activity
VTIME	vertical movements time
VMOVNO	number of vertical movements
